# Unknotting night-time muscle cramp: a survey of patient experience, help-seeking behaviour and perceived treatment effectiveness

**DOI:** 10.1186/1757-1146-5-7

**Published:** 2012-03-15

**Authors:** Fiona Blyton, Vivienne Chuter, Joshua Burns

**Affiliations:** 1 The University of Sydney Medical School/School of Health Sciences, The University of Newcastle, PO Box 127, Ourimbah 2258 NSW, Australia; 2 School of Health Sciences, The University of Newcastle, PO Box 127, Ourimbah 2258 NSW, Australia; 3 Faculty of Health Sciences, The University of Sydney/Institute for Neuroscience and Muscle Research, Sydney Children's Hospitals Network, The Children's Hospital at Westmead, Locked Bag 4001, Westmead NSW 2145, Australia

**Keywords:** Sleep disorders, Quinine, Magnesium, Calf muscle

## Abstract

**Background:**

Night-time calf cramping affects approximately 1 in 3 adults. The aim of this study was to explore the experience of night-time calf cramp; if and where people seek treatment advice; and perceived treatment effectiveness.

**Methods:**

80 adults who experienced night-time calf cramp at least once per week were recruited from the Hunter region, NSW, Australia through newspaper, radio and television advertisements. All participants completed a pilot-tested survey about muscle cramp. Quantitative data were analysed with independent-sample *t*-tests, *Chi *square tests and Fisher's tests. Qualitative data were transcribed and sorted into categories to identify themes.

**Results:**

Median recalled age of first night-time calf cramp was 50 years. Most participants recalled being awoken from sleep by cramping, and experiencing cramping of either calf muscle, calf-muscle soreness in the days following cramp and cramping during day-time. Despite current therapies, mean usual pain intensity was 66 mm on a 100 mm visual analogue scale. Participants described their cramps as being '*unbearable*', '*unmanageable*' and '*cruel*'. One participant stated that '*sometimes I just wish I could cut my legs open*' and another reported '*getting about 2 h sleep a night due to cramps*'. Most participants had sought advice about their night-time calf cramps from a health professional. Participants identified 49 different interventions used to prevent night-time calf cramp. Of all treatment ratings, 68% described the intervention used to prevent cramp as being 'useless' or of 'a little help'. Of 14 participants who provided additional information regarding their use of quinine, eight had a current prescription of quinine for muscle cramp at the time of the survey. None had been asked by their prescribing doctor to stop using quinine.

**Conclusion:**

Night time calf cramps typically woke sufferers from sleep, affected either leg and caused ongoing pain. Most participants experienced little or no relief with current therapies used to prevent muscle cramp. Most people who were taking quinine for muscle cramp were unaware that the Australian Therapeutic Goods Administration withdrew support of quinine for muscle cramp in 2004 due to the risk of thrombocytopaenia. Case-control studies are required to identify therapeutic targets so that clinical trials can evaluate safe interventions to prevent recurrent cramp.

## Background

Muscle cramps are sudden, involuntary, painful contractions of skeletal muscle [1,2]. They are characterised electrically by repetitive firing of motor unit action potentials at rates up to 150 per second [3], more than four times the usual rate in maximum voluntary contraction [4]. Approximately 1 in 3 adults experience night-time cramping of the calf-muscle [5], the most common type and location of cramp [6-8]. Despite the high prevalence of night-time calf-muscle cramps, it is not well understood why some people cramp and others do not. Currently, many interventions are available for preventing night-time calf-muscle cramps (*e.g. *quinine, magnesium, and muscle stretching, strengthening and splinting) but no drug therapy [9] or physical therapy [10] has demonstrated adequate efficacy and safety. No drug therapy is currently approved under the Australian Pharmaceutical Benefits Scheme (PBS) [11] for muscle cramps. In 2004, the Australian Therapeutic Goods Administration (TGA) withdrew approval of quinine sulphate or bisulphate tablets (Quinate or Quinbisul) for muscle cramps due to the risk of thrombocytopenia (decreased platelet count) [12]. Quinine is now a streamlined authority PBS item, subsidised only for use in malaria, and doctors are encouraged by the TGA to ask patients to stop taking quinine for muscle cramp [13].

Management of leg cramps has been described as being frustrating [14] and difficult [15]for clinicians and patients alike. It has been suggested that many people experience no benefit from the treatments prescribed [3,7,16] and many more receive no treatment at all [5,7,15], however these assertions have not been evaluated.

The aim of this study was to explore: (1) the experience of night-time calf cramp; (2) if and where people seek treatment advice; and (3) perceived treatment effectiveness.

## Method

Adults who experienced night-time calf cramp at least once per week in the preceding three months volunteered to participate between August 2010 and March 2011. Muscle cramp was described as *'a sudden, involuntary and painful contraction of muscle that gradually lessens. During cramp, the affected muscle hardens and joints can be forced into unusual positions. In some people, cramp can be brought on by certain movements and/or stopped by stretching the muscle'*. This definition was based on descriptions reported in the literature [1,2,17] and on clinical experience in describing cramping to patients. Exclusion criteria were: dementia, lower limb injury, and conditions known to cause cramps (including, neuromuscular or neurological disease, pregnancy; and haemodialysis). No exclusions were made based on medication use or current treatment of cramps. All participants were proficient in English and ambulant. Participants were recruited from the Newcastle, Central Coast and Hunter Valley regions of NSW, Australia. The study was promoted: on regional television news and radio; in four regional newspapers; in reception areas of a podiatry practice, University clinic and local General Practice, and by contacting local community groups (*e.g. *Lions and Rotary Clubs). The research was promoted as an investigation of the cause of muscle cramp. This limited the potential recruitment of people with conflicts of interest (*e.g. *people affiliated with companies selling treatments for night cramps). Interested community members contacted the principal investigator (F.B.) by phone. All participants consented to participate in accordance with the University of Newcastle Human Research Ethics Committee.

No validated surveys are available for night-time muscle cramping. Prior to commencing the research, a bespoke survey was developed and pilot-tested for content validity by four health professionals with experience in treating night-time muscle cramp, by two patients who experience night-time calf cramp and by two other non-health professionals. During pilot testing each participant was asked to identify any ambiguous questions or statements and to suggest additional questions. Some minor amendments to technical language were made to produce the final survey. The survey comprised questions covering: gender; age; family history of night-time cramp; age of cramp onset; leg/s affected; timing of cramps; usual pain severity of cramp (rated on a 100 mm visual analogue scale where 0 = no pain and 100 = worst pain imaginable); cramp frequency; presence of calf pain during the day/s after cramp; consistency of the pattern of cramping; whether advice had been sought regarding night-time calf cramp (if so, from whom, and the effectiveness of recommended treatments); other treatments tried; whether muscles other than the calf cramp at night (if so, which muscles); and whether muscle cramp also occurs during the day (if so, which muscles and when). Participants were also given the opportunity to add comments about the interventions used and were asked the question: 'What do you think causes your cramps?' A survey was posted to each participant and returned by post or in-person to one of the clinics promoting the research. Participants who reported having used quinine for night-time calf cramps were followed up to check whether or not they were still using quinine for cramps. Participants who were still using quinine were asked how often they take it, if the prescribing doctor was a general practitioner, and if their prescribing doctor had: (1) asked them to stop taking quinine, (2) explained that quinine was no longer approved under the PBS for muscle cramp; (3) discussed the possible adverse effects of quinine.

Quantitative data were analysed using SPSS v.19 (Chicago, IL, USA) to produce descriptive statistics. Continuous data were tested for normality using the Kolmogorov-Smirnov test. The non-normally variable 'age at cramp onset' was described using median and interquartile range (IQR) and was transformed to normal distribution for inferential statistical analysis using the formula reflect and square root insert [18]. Statistical significance of between-group differences were tested with independent-sample *t*-tests for continuous data and either *Chi*-square analysis (with Yates Continuity Correction for 2 by 2 tables) or Fisher's exact test (if a cell in the 2 by 2 table had less than 5 counts) for dichotomous data. Responses to the question 'What do you think causes your cramps' were transcribed and sorted into mutually exclusive and exhaustive categories using emergent coding [19].

## Results

Eighty-three potential participants were sent an information sheet, consent form and survey. Eighty people (47 females and 33 males) volunteered to participate and completed the survey. Age ranged from 37 to 93 years (mean 71; standard deviation [SD] 10). Of the three people who did not return the survey, one 'decided against it', one could not find the time, and one did not respond to follow up phone calls and emails.

### Description of the night-time calf cramps

The usual pain intensity of night-time calf cramp rated on a 100 mm visual analogue scale ranged 29 mm to 100 mm (mean 66; SD 19). There was no statistically significant difference in usual pain intensity of night-time calf cramp between men and women (63 mm vs. 69 mm; mean difference -6 mm; 95%CI: -15 to 2; *t*_77 _= -1.43; *p *= 0.16), or between younger (≤ 64 years, n = 15) and older ( ≥ 65 years, n = 64) people (69 mm vs. 66 mm; mean difference 3 mm; 95%CI: -7 to 15; *t*_77 _= 0.69; *p *= 0. 49. Recalled age of first night-time calf cramp ranged from 9 to 85 years (median 50; IQR 35). 26% of participants reported a known family history of night-time leg cramps.

Of the 74 participants who could recall when cramps typically begin, 45 (61%) recalled usually being asleep when cramps begin. Seventy one (89%) participants reported that cramp could affect either leg. Cramps were mostly reported to occur at irregular times throughout the night (n = 32, 40%), during the middle of sleeping time (n = 24, 30%), within two hours of rising in the morning (n = 10, 12.5%), before falling asleep (n = 6, 7.5%) and within two hours of first falling asleep (n = 6, 7.5%). Two participants (2.5%) were unsure of cramp timing. Calf-muscle soreness or tenderness in the days following cramp was reported to occur sometimes by 32 (40%) participants, most of the time or always by 25 (32%) participants and never or rarely by 22 (28%) participants. People who most of the time or always experienced pain during the days following cramp were more likely than people who never, rarely or sometimes experienced pain during the days following cramp to have experienced more intensely painful night-time calf cramp (75 mm vs. 63 mm; mean difference 12 mm; 95% CI: 3 to 21; *t*_77 _= 2.7; *p *= 0.008), but were no older (n = 15) or younger (n = 64) (*t*_77 _= 0.69; *p *= 0.49).

Thirty-three percent of participants reported experiencing night-time calf cramp most nights per week. Between people who experience calf cramp ≤ 3 nights per week and people who experience calf cramp most nights per week, there was no statistically significant difference in mean usual pain intensity of night-time calf cramp (65 mm vs. 75 mm; mean difference -10 mm; 95%CI: -23 to 2; *t*_77 _= -1.6; *p *= 0. 11) or mean age (71 years vs. 75 years; mean difference -4 years; 95%CI: -11 to 2; *t*_77_; -1.38; *p *= 0. 17). 63% of participants reported frequency of night-time calf cramp as being inconsistent.

Sixty-eight (85%) participants also reported experiencing night cramp in muscles other than the calf. 61 (76%) participants reported experiencing muscle cramp during the day while awake. Locations of night and day cramps are listed in Table 1. Participants who experienced day time muscle cramp were no more likely to experience night-time muscle cramp of muscles other than the calf (*p *= 0.68, Fisher's exact test) and participants who experienced day time calf cramp were no more likely to experience more frequent night-time calf cramp (*p *= 0.50, Fisher's exact test). Day cramps were reported to occur: at rest while sitting (n = 44, 72%); while lying down (n = 23, 38%); during physical activity other than walking or climbing stairs (n = 21, 34%); while standing (n = 14, 23%); while walking (n = 9, 15%); while climbing stairs (n = 4, 7%); and at other times (n = 7, 11%).

**Table 1:** Location of night and day time cramping

Location of cramp	During the night, n of participants	While awake during the day, n of participants
Calf	80	37

Foot	49	32

Front of lower leg	28	8

Hand	25	32

Inner thigh	25	14

Back of thigh	23	13

Front of thigh	16	5

Arm	9	7

Buttock	4	1

Other	10	11

### Treatment advice

Sixty of the 80 participants had sought advice about their night-time calf cramps. All reported sources of advice are listed in Table 2. All 10 participants who reported cramping most nights per week had sought advice, but the difference to those who reported cramping three or less night per week was not statistically significant (100% vs. 71%; Fisher's Exact Test *p = *0.58). Cramp pain intensity was not associated with seeking advice (*t*_77 _= 0.58; *p *= 0.56) and women were no more likely than men to have sought advice about their night-time calf cramp from any source (*χ*^2 ^[1, n = 80] = 1.4, *p *= 0.24, p*hi *= 0.16). Participants who had sought advice were six years older on average than those who had not (73 years vs. 67 years; mean difference 6 years; 95% CI: 1 to 11; *t*_78 _= -2.45; *p *= 0.02). Participants who had sought advice about their night-time calf cramp over the internet were 13 years younger on average than people who had not (61 vs. 74 years; mean difference 13 years; 95%CI: 4 to 22; *t*_58 _= 2.78; *p *= 0.007).

**Table 2:** Sources of advice

	Number of participants
General practitioner	51

Pharmacist	14

Podiatrist	8

Medical specialist	7

Family member or friend who is not a health professional	6

Physiotherapist	5

Naturopath	5

Internet	4

Chiropractor	3

Traditional Chinese Medicine practitioner	2

Bowen therapist	1

Massage therapist	1

Health food herbalist	1

Health food shop assistant	1

All participants had tried to treat their night-time calf muscle cramps. All reported interventions and ratings of perceived effectiveness are listed in Table 3. Only two participants rated a treatment used to prevent cramp as being 100% effective (both ratings for quinine). Of all 197 ratings of preventative interventions, 132 (67%) described the interventions as being 'useless' or 'a little help'. Only 19 (10%) described preventative interventions as being very helpful or 100% effective. Nine of these 19 ratings were for quinine. Overall, interventions used during cramp were perceived as being more effective than interventions used to prevent cramp.

**Table 3:** Reported interventions and perceived effectiveness

Intervention	Number of users	Perceived effectiveness					
		
		Useless	A little help	Quite helpful	Very helpful	100% effective	Unrated
**During cramp to reduce pain**							

Getting out of bed to stand or walk*	77	4	21	19	27	5	1

Stretching calf*	75	20	22	20	12	1	

Massage*	69	6	42	13	7		1

Heat application	4			3	1		

Running on the spot	1		1				

**To prevent cramp**							

Magnesium	46	12	16	11	5		2

Water, drinking more*	36	11	19	5			1

Stretching calf during day or before bed*	24	7	11	2	1		3

Massage during day*	21	5	11	5			

Quinine	18	1	1	7	7	2	

Crampeze tablet/capsule	13	5	6	2			

Tonic water	5	1	2	2			

Gatorade/poweraid	4	1		3			

Salt	3		1		2		

Crampeze cream	2		1	1			

Vitamin B	2		1	1			

Akineton *(biperiden)*	2	1	1				

Hamstring stretching	2	1	1				

Lyrica *(pregabalin)*	1				1		

Schuessler tissue salts (homeopathic preparation)	1				1		

Camphor in bed	1			1			

Cramp away (homeopathic preparation)	1			1			

Exercise and stretching with personal trainer	1			1			

Filtered water	1			1			

Homeopathic drops containing ginkgo	1			1			

Iron tablets	1			1			

Japanese green tea	1			1			

Acupuncture	1		1				

Calcium	1		1				

Epsom salt bath	1		1				

Glucosamine	1		1				

Minerals	1		1				

Panadol osteo	1		1				

Tegretol (*carbamazepine)*	1		1				

Shaking of legs during shower	1		1				

Aspirin	1	1					

Fish oil	1	1					

Mandopar (*levodopa and benserazide)*)	1	1					

Multi vitamins	1	1					

Potassium	1	1					

Vitamin E	1	1					

Vitamins	1	1					

Zinc	1	1					

Bananas	1						1

Oranges	1						1

*Participants were asked whether they had tried interventions identified with an asterisk. Non-asterisked interventions were identified by participants when asked whether they had tried any other interventions

Interventions are ordered from most to least commonly reported. Where a particular treatment is reported as commonly as another, interventions are ordered from perceived most to least effective, then in alphabetical order.

Participants who had used quinine for muscle cramp (n = 18) were more likely than people who had not used quinine for muscle cramp to experience more frequent night-time calf cramp (28% ≥ 4 night per week vs. 8% ≥ 4 night per week *p *= 0.04, Fishers exact test) but were no more likely to: be female (61% vs. 58%, *χ*^2 ^[1, n = 80] = 0.00, *p *= 1.00, phi = 0.03); be older (mean 74 years vs. 70 years, *t*_78 _= -1.59; *p *= 0.12); experience more painful night-time calf cramp (mean 67 mm vs. 66 mm, *t*_77 _= -0.23; *p *= 0. 82); and have earlier onset of night-time calf cramp (mean 45 years vs. 49 years, *t *= [71] = 0.63; *p *= 0. 53).

### Use of quinine

Of the 18 participants who reported having used quinine for night-time calf cramp, 14 responded to a follow up telephone call to provide more information. Six participants had stopped taking quinine between 18 months and 8 years ago. Of these, three participants were advised by their General Practitioner (GP) to stop using quinine due to safety concerns. Of the remaining three, one was notified of the associated risks by a chemist, one stopped experiencing cramps so discontinued using quinine, and one discontinued taking quinine to try a non-prescription treatment.

Eight participants reported current use of quinine for cramps. All eight received prescription from their GP. Three reported taking quinine daily, one reported taking quinine every second night, and four reported taking quinine only when cramps were particularly bad, ranging from 'once or twice a week' to 'once per month, on average'. None of the eight had been asked by their GP to stop taking quinine. Only two had been told by their GP that quinine was not covered under the PBS. Six participants could not recall being notified of possible adverse effects by their GP. Of the other two, one reported being told that it could 'affect the heart', and one recalled being told that quinine wasn't good for him, but the participant was not sure why.

### Qualitative data

Of the 62 participants who responded to the open-ended question 'What do you think causes your cramps?' 20 responded that they did not know. Eleven participants thought cramps could be due to the position in which they sleep and movements from this position (for example, '*if I straighten my legs quickly*'). One participant reported a cramp triggered by an event in a dream where they suddenly needed to run. Nine participants thought that cramps could be due to poor circulation to the lower limbs. Five participants thought cramps could be due to unusually high levels of physical activity during the day. One other participant thought cramp could also be due to unusually low levels of activity. Another participant suggested night cramp was due to holding legs in '*one cramped position*' on a bus trip during the day. Four participants thought that cramp could be due to lack of salt. One participant added '*a doctor said this 25 years ago'*.

Three participants associated night cramp with nocturia, but disagreed as to whether cramp was caused by '*a full bladder'*, or moving the legs on waking. Two participants thought cramps could be due to '*lack of water'*, or '*dehydration*'. Other suggestions included '*uric acid'*, '*age*', '*tiredness*', '*cold*', '*hereditary*', '*after IV chemotherapy'*, '*night sweats'*, '*hypoglycaemia*', '*chocolate*', '*alcohol'*, '*peripheral neuropathy'*, '*some connection with the perception of cold' *or '*another nasty complication of my diabetes'*. One participant commented that cramps '*started when I moved from England at 67 [years of age]*' and are '*worse in summer after playing bowls*'. Another participant, after stating that she had 'no idea' what causes the cramps, commented '*it is cruel. Sometimes I just wish I could cut my legs open*'. Cramps were described as 'unbearable' and 'unmanageable'. One participant stated they were '*getting about 2 h sleep a night due to cramps' *and another said that they '*can't do anything after cramps due to fear of getting another one*'.

## Discussion

Most participants recalled being awoken from sleep by cramps and cramps occurring at irregular times or in the middle of sleeping time. These findings suggest that night-time muscle cramping may profoundly affect quality of sleep. Most participants also reported experiencing calf-muscle soreness in the days following cramp. There are anecdotal reports in the literature of discomfort persisting for hours following night-time muscle cramp [15]. This is the first study to demonstrate the prevalence and lengthy duration of ongoing pain.

Mean usual pain intensity of night-time calf cramp was 66 mm on a 100 mm visual analogue scale and most participants also experienced night-time muscle cramping of other muscles and day time muscle cramping. The severity and high frequency of cramps reported by participants are despite current therapies. As most participants reported experiencing some help from treatment, it is expected that frequency and severity of cramp would be higher if current treatments were withdrawn.

Median reported age of first cramp (50 years) in this sample was younger than previous reports in the literature. Of 86 respondents to a United Kingdom (UK) postal survey who reported suffering from rest cramps, the mean age of cramp onset was 60 years (95%CI: 57 to 63) [15]. This result is reflected by a second UK survey of 182 people over 65 years of age who experienced leg muscle cramp. 80% of participants reported cramp onset after 55 years of age [7]. The younger age of cramp onset in the present sample might reflect the inclusion of adults of all ages or differences in exposure to cramp precipitants (*e.g. *climate) between the UK and Australia. Indeed one participant stated that cramps '*started when I moved from England at 67 [years of age]*', although this could be coincidental.

Advice-seeking behaviour in our sample was more common than in previous reports in the literature. In a UK survey of 182 people over 65 years of age who experienced leg muscle cramp, only 40% sought advice from their General Practitioner about their cramp (contrast to 63% in our sample) [7]. This is similar to the results of a similar survey in England of people randomly selected from a General Practitioner's Register [15]. Interestingly, of all participants who had not reported cramp to a General Practitioner, 31% described their cramps as being very distressing or a major nuisance [15]. Despite the present finding of more common advice seeking, only two participants reported achieving cramp prevention with treatments recommended. Both reports related to quinine, which doctors in Australia are now discouraged from prescribing for muscle cramps.

The most commonly reported treatment used to prevent recurrent cramp was magnesium supplementation (used by 58% of participants). Most participants perceived the magnesium supplements as being 'useless' or only 'a little help'. This is consistent with randomised trials that found that, while magnesium supplementation might benefit women experiencing leg cramps during pregnancy [20], it is no more effective than placebo for night-time cramp [21] or chronic persistent leg cramp [17]. The effectiveness of magnesium for skeletal muscle cramps is currently being reviewed under the Cochrane Neuromuscular Disease Group [22].

Interestingly, in the trial of magnesium citrate for chronic persistent leg cramps [17], about half of people allocated to the placebo group perceived that placebo had helped. For all interventions reported in the present study, it is not possible to disentangle any placebo effect from true intervention effects. It is possible that the placebo effect accounts for some, if not all, of the perceived effectiveness of some interventions.

Interpretation of some findings is constrained by limitations of the survey. For example, people who reported stretching the calf muscles were not asked to demonstrate how they performed the stretch. On discussion with some participants, it became clear that some stretching techniques were inappropriate, for example, actively plantarflexing the foot to stretch the calf muscle. While this limits the interpretability of effectiveness ratings for calf stretching in this survey, it clearly demonstrates the need for health practitioners to carefully demonstrate and check stretching techniques when advising their patients to stretch. A second limitation of the survey is the potential for recall bias when nominating interventions used now and in the past. Participants were asked to list all interventions that they had used for night-time muscle cramp. In addition, participants were specifically asked whether they had used certain interventions. These are marked with asterisks in Table 3. Direct questioning may have improved recall and led to an increased response rate for these interventions.

The most commonly suggested cause of cramp was sleeping position and movements from this position. Participants who identified the particular movements that induced cramp described plantarflexion of the ankle joint as the trigger. To prevent calf cramp, one participant described sleeping with a foot board at the base of their bed to prevent ankle joint plantarflexion and others described actively dorsiflexing the ankle joint when the early symptoms of cramp appeared. Avoidance of ankle plantarflexion has been described in the literature as a potential treatment to prevent recurrent cramp [23-26]. Suggestions include using pillows [25,27] or a foot board [24] to resist ankle plantarflexion, and sleeping prone with feet overhanging the end of the bed [24]. None of these approaches have been evaluated in clinical trials and some might not be practical due to propensity to reposition during sleep [27]. Use of a dorsiflexion night-splint would offer a more controlled and sustained dorsiflexion, yet this has not been evaluated or even suggested, to our knowledge.

## Conclusions

Night time calf cramps typically awoke sufferers from sleep, affected either leg and caused ongoing pain. Most participants experienced little or no relief with current therapies used to prevent muscle cramp. Most people who were taking quinine for muscle cramp were unaware that the Australian Therapeutic Goods Administration withdrew support of quinine for muscle cramp in 2004 due to the risk of thrombocytopaenia. Case-control studies are required to identify therapeutic targets so that clinical trials can evaluate safe interventions to prevent recurrent cramp.

## Abbreviations

CI: Confidence interval; GP: General practitioner; IQR: Interquartile range; N: Number; TGA: Therapeutic good administration; PBS: Pharmaceutical benefits scheme

## Competing interests

Fiona Blyton and Vivienne Chuter declare that they have no competing interests. Joshua Burns has receives research support from the NHMRC (National Health and Medical Research Council of Australia, Fellowship #1007569 and Centre of Research Excellence #1031893), NIH (National Institutes of Neurological Disorders and Stroke and Office of Rare Diseases, #U54NS065712), Australian Podiatry Education and Research Foundation, Charcot Marie Tooth Association, Muscular Dystrophy Association, CMT Association of Australia.

## Authors' contributions

FB and JB designed the study methods. FB designed and validated the surveys, screened potential participants for inclusion, collected data, performed statistical analyses and drafted the final manuscript. VC provided comment on the final manuscript. JB provided comment on the surveys, draft and final manuscript. All authors read and approved the final manuscript.
